# Bovine Udder Health: From Standard Diagnostic Methods to New Approaches—A Practical Investigation of Various Udder Health Parameters in Combination with 16S rRNA Sequencing

**DOI:** 10.3390/microorganisms11051311

**Published:** 2023-05-17

**Authors:** Verena Rötzer, Jasmin Wenderlein, Anna Wiesinger, Felix Versen, Elke Rauch, Reinhard K. Straubinger, Eva Zeiler

**Affiliations:** 1 Faculty of Sustainable Agriculture and Energy Systems, University of Applied Science Weihenstephan-Triesdorf, 85354 Freising, Germany; felix.versen@hswt.de (F.V.); eva.zeiler@hswt.de (E.Z.); 2 Chair of Animal Welfare, Ethology, Animal Hygiene and Animal Husbandry, Department of Veterinary Sciences, Faculty of Veterinary Medicine, LMU Munich, 80539 Munich, Germany; rauch@lmu.de; 3 Chair of Bacteriology and Mycology, Institute for Infectious Diseases and Zoonoses, Department of Veterinary Sciences, Faculty of Veterinary Medicine, LMU Munich, 80539 Munich, Germany; jasmin.wenderlein@lmu.de (J.W.); anna.wiesinger@micro.vetmed.uni-muenchen.de (A.W.); r.straubinger@lmu.de (R.K.S.)

**Keywords:** mastitis, diagnostics, conventional culturing, 16S rRNA gene amplicon sequencing, bacterial community, udder health, scoring

## Abstract

Bovine udder health is an important factor for animal wellbeing and the dairy farm economy. Thus, researchers aim to understand factors causing mastitis. The gold standard for diagnosing mastitis in cows is the conventional culturing of milk samples. However, during the last few years, the use of molecular methods has increased. These methods, especially sequencing, provide a deeper insight into the diversity of the bacterial community. Yet, inconsistent results regarding the mammary microbiome have been published. This study aimed to evaluate the udder health of eight dairy cows at seven days postpartum with the standard methods in veterinary practice. Additionally, swabs from the teat canal and milk samples were analyzed using 16S rRNA gene amplicon sequencing. The sensitive low-biomass milk samples displayed only a few contaminations even though they were sampled in a field environment. In healthy udders, no bacterial communities were detected by the bacterial culture nor the 16S rRNA gene amplicons. The results from the standard examination of the cows, the cell count, and the bacteriological examination were comparable with the results from 16S rRNA gene amplicon sequencing when cows displayed subclinical or latent mastitis. Besides the pathogen detected in bacterial culturing, a second bacterial strain with low but significant abundance was detected by sequencing, which might aid in the understanding of mastitis incidence. In general, molecular biological approaches might lead to promising insights into pathological events in the udder and might help to understand the pathomechanism and infection source via epidemiological analyses.

## 1. Introduction

Bovine udder health is an important factor in dairy farms as diseases of the bovine udder are detrimental to the cow’s wellbeing [1]. They further influence milk quality [2], increase the risk of developing antimicrobial resistance [3], and have an economic impact [4]. The dry period is a sensitive phase in which the udder undergoes morphological changes by involution as the cow moves closer to parturition [5]. During this stage of non-lactation and especially in the two-week period after cessation of milking and during colostrogenesis, the udder is susceptible to intramammary infections [6]. For many decades, the conventional culturing (CC) of milk samples has been a quick and affordable method to identify pathogenic bacteria involved in mastitis and to further identify a fitting antibiotic to fight the pathogens [7,8,9]. Nevertheless, there are some limits of CC pertaining to the sensitivity of the method. In general, only a small fraction of all cow-inhabiting bacteria is cultivable [10,11], while most known mastitis-causing bacteria are detected by CC. In contrast, dead bacteria, growth-inhibited bacteria, and other pathogens causing intramammary infections cannot be detected by CC [12]. Consequently, the result “no bacterial growth” is frequently observed as a result of CC testing using milk samples. The proportion of negative results from CC is around 20–30% in milk samples from cows with clinical mastitis, while that in cases with subclinical mastitis the proportion is slightly higher [13]. Therefore, during the last decade, several molecular techniques such as PCR and next-generation sequencing have been introduced to enlarge the spectrum of methods for mastitis diagnostics. These new approaches provide better sensitivity in detecting bacteria and especially bacterial DNA in milk in comparison to CC [12,14,15,16]. Due to the increase in knowledge and the availability of modern analysis techniques, new perspectives have emerged regarding the colonization of microbial communities in various organs and regions of the body [17]. The so-called “microbiome” has been the focus of research in recent years. The microbiome is defined as the totality of microorganisms, their genomes, and the surrounding environmental conditions, which exist in a defined habitat [18]. This study focuses on one group of the microbiota, the bacterial community, as most of the previously known mastitis-causing pathogens represent bacteria. Recently, the mammary gland has been proposed to harbor a diverse microbiome. However, this matter is discussed controversially [19]. There are both supporters and objectors of the hypothesis of the presence of a natural community of microbes within the mammary gland [19,20,21,22]. Furthermore, the term milk microbiota is not yet clearly defined regarding its origin from either only the milk or the mammary gland. Nevertheless, to understand the origin of the microbiome in the cow’s healthy or diseased udder, it is important to differentiate between the milk, the skin at the teat’s tip, the teat canal, and the glandular body. Three origins of the microbiota in the udder microbiome have been described: the internal upper part of the mammary gland (i.e., gland or teat), the extramammary outsides (i.e., skin), and the environment (i.e., feces or bedding) [21,23]. The upper part of the mammary gland is the origin of milk production, and a few studies focused especially around this location (i.e., teat or gland cistern) [24,25,26], whereby reasonable results could only be obtained by direct sampling from the gland cistern [19]. However, most studies have sampled outside the mammary gland once the milk has been expressed [17]. The studies by Hiitiö et al. [24] and Metzger et al. [25] are the only studies that sampled via a direct method by pricking a needle into the gland cistern. Other researchers have sequenced material from the teat canal, a separate compartment within the mammary gland with possibly different colonization [27,28,29]. In general, the following milk- and udder-associated microbiota have been described: *Pseudomonas* spp., *Staphylococcus* spp., *Streptococcus* spp., Lachnospiraceae, *Corynebacterium* spp., *Bacteroides* spp., and *Enterococcus* spp. [17]. Many of these bacteria are common inhabitants of the bovine skin or the environment [19,21,23] and many of the bacteria, which potentially compose the milk microbiota of healthy mammary glands, have the potential to induce mastitis [19]. Apart from arguments supporting the theory of a milk or udder microbiome, there are convincing arguments opposing this theory. This matter has been reviewed in great detail by Rainard et al. [19]. Reasons raised against the existence of a microbiome in the mammary gland are the inability of the innate and adaptive immune system of the mammary gland to adapt to physiological colonization with bacteria or the lack of an entero-mammary pathway in ruminants. Furthermore, milk is described as a nutrient-rich medium that supports the growth of bacteria. Additionally, the lack of a mucus shield in the epithelium of the mammary gland, which would limit the direct contact of bacteria with the epithelium, is an argument against the possible colonization of the udder with microbiota [19]. Further arguments against a bovine udder microbiome have been discussed elsewhere [20]. One main reason why some studies describe a bovine udder microbiome might arise from the difficulty in handling low-biomass or sterile samples in next-generation sequencing. As these methods are extremely sensitive and low amounts of DNA can be amplified, contaminations are at great risk to produce false-positive results. A prime example is the now mostly proven inexistence of a fetal microbiome in utero [30]. Researchers must therefore critically question their data, especially when handling low- or no-biomass samples, and as such, sterile working and published standards for microbiome studies should be applied [31].

The present study aimed to investigate various udder health parameters in combination with next-generation sequencing of 16S rRNA gene amplicons. Thereby, standards for microbiome studies have been applied [31] such as the inclusion of various negative controls of the farm environment, all fluids used during sampling and DNA extraction, and a mock community. In a small group of lactating Simmental cows (*n* = 8), the condition of the udder health in the first week postpartum was examined. At the time of dry-off, the cows had received neither intramammary nor systemically applied antimicrobials, but rather an internal teat sealant. The internal teat sealant consisting of alkaline bismuth nitrate was applied through the teat canal with an injector after the last milking. To comprehend the state of the udder’s health, various parameters were recorded. The objective of this pilot study was to evaluate the benefit of sequencing as a new investigation method in comparison with the current gold standard under the highest possible and practicable levels of decontamination in a field environment.

## 2. Materials and Methods

### 2.1. Ethical Committee Approval

The presented study was approved by the Ethical Committee of the Faculty of Veterinary Medicine of LMU Munich (reference number 309-16-05-2022).

### 2.2. Animals and Farm Conditions

For this study, eight Simmental dairy cows from a farm located in Upper Bavaria were chosen. The cows were kept in a cubicle stall consisting of four individual pens according to their health and lactation status. The bedding material provided was either a straw mattress in the calving and sick pen or deep-bedded cubicles with straw in the pen for lactating cows and dried-off cows. The cows received a total mixed ration depending on their production level and were milked two times a day. The dairy farm consisted of 94 lactating cows with an average annual milk production of 9336 kg per cow and an average somatic cell count (SCC) of 211,000 cells per mL according to the Landeskuratorium der Erzeugerringe für tierische Veredelung in Bayern e.V. (LKV) data analysis in 2022. The average lactation number of the herd was 4.3. The animals were selected based on having the following criteria: pluriparity, dry-off phases without antibiotics but with an internal teat sealant, a calving event in the timeframe of the experiment, and no complications during birth. The selected animals calved during the test period and were investigated on the seventh day in milk (DIM). The investigation took place during the normal morning milking in the milking parlor of the farm, which is constructed as a double-six herringbone. The investigation included the following steps: scoring, sample collection for the CC, and thereafter sampling for 16S rRNA gene amplicon sequencing.

### 2.3. Scoring

The scoring was conducted as a combination of a general examination and a further examination of the udder including a sensory examination of the milk secretion. With the collected information, the udder health status of the cows was determined. Table 1 provides an overview of the examinations conducted for the scoring, the examination methods, and the evaluation key according to the sources used.

In the general examination, the body temperature was measured rectally using the veterinary thermometer SC 12 (Scala Electronic GmbH, Stahnsdorf, Germany). The respiratory movements at the costal arch and flank were counted and the rumen score, modified according to Donat et al., was determined [33,36,37].

The rumen scoring system is a modified three-stage score to visually evaluate the rumen filling of the animals and as a result, the associated dry matter intake. The evaluation took place before milking in the morning with the examiner standing in a distro-lateral position to the cow. The used grading scheme is provided in Supplementary Table S1.

An udder examination was carried out for each animal [32]. Thereby, all udder quarters were visually examined and palpated, and a sensory examination of the quarter milk samples was conducted. During the visual examination, the hygiene of the udder and the lower leg was assessed by standing behind the cow using an udder hygiene scoring chart with a four-stage scoring scheme [7,34,35] presented in Supplementary Table S2. This tool allows macroscopic assessment regarding the degree of manure contamination of the examined cows. Animals with an udder hygiene score of “1” or “2” are considered “clean” and cows with a score of “3” or “4” are considered “dirty” [7]. The palpation consisted of an examination of the udder skin and the glandular body including the glandular cistern and the teats. First, each udder quarter was palpated superficially with both hands. Thereafter, each udder quarter was palpated deeply to assess the texture, condition, and temperature. For the macroscopic sensory examination of the milk, the first milk streams were milked into a pre-milking cup whereby the functionality of each quarter was checked. Qualitative variations such as color, smell, consistency, and additions in the quarter milk samples were examined. The classification was made according to the scheme in Supplementary Table S3.

### 2.4. Udder and Milk Sample Collection

Before the first milking in the morning, all samples from the udder and milk were collected. The order of sampling was chosen according to the sensitivity of the method: conventional quarter milk samples (CMS), swabs of the teat canal (MS), and milk from the gland cistern (MS). Before CMS were collected, teats were thoroughly cleaned and disinfected with 70% ethanol swipes. The first streams of milk were discarded. Thereafter, CMS were collected by manual milking into prepared tubes, which were set up by the company bovicare (bovicare GmbH, Bernau, Germany) according to the guidelines of the German Society of Veterinary Medicine (DVG) [9]. Afterward, the samples for 16S rRNA gene amplicon sequencing were collected. Tubes used for the collection were placed as far as possible from the milking stand to reduce contamination of the collection material. One person exclusively handled the sterile transfer of swab samples into tubes prefilled with 600 µL DNA Stool Stabilizer (Invitek Molecular GmbH, Berlin, Germany) and milk samples into 50 mL Flacon Tubes (Sarstedt AG und Co. KG, Nümbrecht, Germany). To minimize contamination, gloves were disinfected with 70% ethanol after each step, and DNA Away was used (Molecular BioProducts Inc., San Diego, CA, USA). Another person collected the samples from the udder, whereby gloves were changed after every teat and disinfected with 70% ethanol before sample collection. First, the teat tips were again thoroughly cleaned with 70% ethanol swipes; thereafter, a sterile swab (2.3 mm × 150 mm; CLS Medizintechnik und Vertrieb, Krebeck, Germany) was used to collect the teat canal sample (TCS). The swab was moistened using a sterile 0.9% sodium chloride solution (B. Braun SE, Melsungen, Germany), and the tip was carefully inserted around 5 mm into the distal end of the teat canal and gently moved to sample the teat canal mucosa before removal. The swab was immediately placed in tubes (Eppendorf SE, Hamburg, Germany) prefilled with 600 µL DNA Stool Stabilizer (Invitek Molecular GmbH). Teat tips were again thoroughly cleaned with 70% ethanol and an atraumatic plastic milking tube (3.3 mm × 63 mm; Bovivet WDT eG, Garbsen, Germany) was inserted into each teat canal. By adapting a sterile 20 mL syringe via the Luer lock system to the milking tube, and 45 mL of milk was obtained from the gland cistern. The milk samples (MS) were thereafter transferred into sterile 50 mL Falcon tubes and flash-frozen on dry ice. All samples obtained for the 16S rRNA gene amplicon sequencing (i.e., TCS and MS) were stored on dry ice for transportation to the Chair of Bacteriology and Mycology of LMU Munich and stored at −20 °C until further sample processing. After sampling, the animals were routinely milked, and teats were dipped before leaving the milking parlor.

### 2.5. Conventional Bacteriological Culturing and Somatic Cell Count

The CMS were sent to a laboratory (bovicare GmbH) for bacteriological examination according to the guidelines of the DVG and for SCC determination. In short, milk samples were cultured on blood agar at 37 °C for 24–48 h as well as on a selective culture medium. The differentiation of the various mastitis pathogens was based on the morphology of the colonies, Gram staining, and the formation of hemolysis zones [9]. The SCC was counted using a fluorometric method with the Fossomatic 5000 (Foss GmbH, Hamburg, Germany). Based on the results of the bacteriological examination, “no abnormality detected (NAD)” and “aseptic” were summarized as “negative”.

### 2.6. Evaluation of the Condition of the Udder Health

Based on the results of the bacterial culturing and the SCC of the quarter CMS, the quarters were categorized according to the scheme in Table 2 [38].

Based on all the results collected, the animals were classified based on the previous cytological and bacteriological evaluation on the quarter level and their clinical signs. The classification resulted in their allocation as either healthy, displaying latent infection, subclinical mastitis, or acute clinical mastitis [38]:

- Healthy animals in terms of the udder displaying normal secretion. The udder quarters display no external pathological changes; the milk is free of pathogenic microorganisms (“negative”) and has a normal average cell count (≤ 100,000 cells/mL).
- In latent infection, pathogens are detected without an increase in the somatic cell count (≤ 100,000 cells/mL).
- Subclinical mastitis is described as an inflammation of the udder without externally visible clinical signs. However, the average SCC of the milk is increased (> 100,000 cells/mL) and mastitis pathogens can be detected.
- Animals with acute clinical mastitis display inflammation on the udder, such as increased temperature and swelling. The milk is macroscopically altered, and the animals often show fever. Mastitis pathogens and an increased SCC are detectable (> 100,000 cells/mL).

### 2.7. Data Analysis Scoring and CMS

The collection and processing of the raw data were performed using Microsoft Excel (Microsoft Office Professional Plus 2016, Microsoft Corporation, Redmond, Washington). Further analyses such as the calculation of averages and medians were carried out using descriptive analyses in IBM SPSS Statistics Version 26 (IBM Deutschland GmbH, Ehningen, Germany).

### 2.8. DNA Extraction—Teat Canal Sample

The 2.0 mL tubes containing TCS and 600 µL of DNA Stool Stabilizer (Invitek Molecular GmbH, Berlin, Germany) stored at −20 °C were thawed at 4 °C. Tubes were then vortexed, and TCS swabs were transferred into a new 2.0 mL tube (Eppendorf SE, Hamburg, Germany). This tube containing only the TCS swab was then centrifuged at 10,000× *g* for 10 s, and any fluid available thereafter was transferred to the tube containing the DNA Stool Stabilizer (Invitek Molecular GmbH). The sample was then mixed, and 100 µL of each TCS was transferred in a new 2.0 mL tube (Eppendorf SE) where all TCS samples from the four udder quarters (four udder quarter suspension, FUQS) of a single cow were pooled. This FUQS of the TCS was then transferred into 2.0 mL lysing matrix tube E (MP Biomedicals, Eschwege, Germany) and homogenized using the FastPrep-24™ device (MP Biomedicals) with three cycles of 6.5 m/s for 40 s, adding dry ice for every cycle. Afterward, 300 µL of Incubation Buffer (D920B-C, Promega GmbH, Walldorf, Germany), 200 µL of Lysis Buffer (included in AS1290, Promega GmbH), and 30 µL of Proteinase K (included in AS1290, Promega GmbH) were added. The tubes were then vortexed and centrifuged for 10 s at 10,000× *g*. Next, samples were incubated at 56 °C and 350 rpm for a minimum of two hours on the shaker. After two hours, 5 µL of 10 mg/mL RNase A (Thermo Fisher Scientific Inc., Waltham, MA, USA) was added, and the samples were incubated for another 20 min at 37 °C and 350 rpm on a shaker. Thereafter, 300 µL of Lysis Buffer (Promega GmbH) was added to each sample, and the vortexing and centrifugation for 10 s at 10,000× *g* were repeated. Similar to the TCS negative controls of the DNA stool stabilizer, 0.9% sodium chloride used to wet the swabs, 75 µL ZymoBIOMICS Microbial Community Standard (Zymo Research Europe GmBH, Freiburg, Germany) as a mock community (MC), and negative controls testing the reagents and cartridge of MaxWell 16 MDx (Promega GmbH) were extracted starting with the homogenization step in the FastPrep-24™ device (MP Biomedicals, Eschwege, Germany). A total of 700 µL of TCS and the negative controls were transferred to the MaxWell 16 LEV Blood DNA Kit cartridges (included in AS1290, Promega GmbH), and the automatized DNA purification was conducted using the Maxwell 16 MDx (Promega GmbH). The samples were eluted in 60 µL Elution Buffer (included in AS1290, Promega GmbH). Purified DNA content was measured using the BioPhotometer (D30, Eppendorf SE) and afterward stored at −30 °C. As measurements of milk and swab samples with the BioPhotometer displayed low DNA amounts, the DNA content was reevaluated using gel electrophoresis. Therefore, 5 µL of DNA from the samples was mixed with 2 µL of loading dye (Thermo Fisher Scientific Inc.). This mixture was then pipetted into the well of a 2% agarose gel. Five microliters of a 1 kB DNA Ladder (New England BioLabs GmbH, Frankfurt am Main, Germany) was used as a control. An Owl Horizontal Electrophoresis System (Thermo Fisher Scientific Inc.) was used to perform agarose gel electrophoresis of nucleic acid (Thermo Fisher Scientific Inc.). The chamber was filled with 1X TAE buffer (pH: 8.5; AppliChem GmbH, Darmstadt, Germany). PowerPac Power Supply (BioRad, Hercules, CA, USA) was applied to the chamber and run with 7.5 V/cm. Thereafter, a DNA gel electrophoresis image was taken using UVP GelStudio (Analytik Jena GmbH, Jena, Germany) with the UV light switched on. Finally, a total of 20 µL of extracted DNA was shipped to Eurofins Genomics (Eurofins Genomics GmbH, Ebersberg, Germany) where the sequencing of the 16S rRNA gene amplicons was conducted.

### 2.9. DNA Extraction—Cisternal Milk Samples

Cisternal MS with a content of 45 mL stored at −80 °C were thawed overnight at 4 °C. Samples were then prepared according to the protocol provided by Siebert et al. [39]. First, 3.0 mL 0.5 M EDTA and 2.0 mL TBE buffer were added to the CMS, which were mixed carefully. Thereafter, samples were centrifuged at 13,000× *g* at 4 °C for 20 min to divide the skim milk and milk fat fraction. The milk fat fraction was removed, and the skim milk was reduced to 1 mL by removing the supernatant and carefully mixed to resuspend the pellet. This suspension was then transferred into a 2.0 mL tube (Eppendorf SE, Hamburg, Germany) and centrifuged at 16,000× *g* for one minute at room temperature. The supernatant was discarded until only 400 µL remained. A total of 100 µL was removed and transferred to a new 2.0 mL tube (Eppendorf SE). All udder quarters of one cow were then pooled to a volume of 400 µL. This FUQS was then transferred into a 2.0 mL lysing matrix tube E (MP Biomedicals) and homogenized using the FastPrep-24™ device (MP Biomedicals) as described for TCS. All further steps were conducted according to the TCS described above.

### 2.10. Bacterial 16S rRNA Gene Sequencing

Both samples (i.e., TCS and MS) were sequenced by Eurofins Genomics (Eurofins Genomics GmbH) by targeting the hypervariable V3/V4 region of the 16S rRNA gene (primers: 5′-CCT-ACG-GGN-GGC-WGC-AG-3′ and 785R: 5′-GAC-TAC-HVG-GGT-ATC-TAA-TCC-3′ [40]). The DNA concentration was reevaluated using a fluorometric method and a fragment analyzer at Eurofins Genomics GmbH. The final pool was sequenced on the Illumina MiSeq platform in paired-end mode (2 × 300 bp), using the MiSeq Reagent Kit v3 (Illumina Inc., San Diego, CA, USA).

### 2.11. Data Analysis Microbiome Data

The 16S rRNA gene amplicon sequencing data were analyzed as described previously [41]. In short, the “Integrated Microbial Next-Generation Sequencing” (IMNGS) platform [42] based on UPARSE [43] was used to obtain both operational taxonomic units (OTUs) and denoised zero-radius operational taxonomic units (zOTUs) for different parts of the analysis. Both the OTU and zOTU tables are provided in Supplementary Tables S4 and S5. The taxonomy was refined using SILVA SSU 138.1 [44], EzBiocloud 2021.07.07 [45], and LPSN ([46] accessed on the 16.01.2023) according to the nomenclature provided by Oren and Garrity [47]. The sequences were realigned and phylogenetic trees were constructed anew using the neighbor-joining method available on the software MEGA 11.0.13 [48]. All downstream analyses were carried out using Rhea [49], a modular pipeline for the microbial profiling of 16S rRNA gene amplicon sequencing data, in an R programming environment (R 3.6.3, R Foundation for Statistical Computing, Vienna, Austria) as already described [41]. Data were visualized using Illustrator CS6 version 16.0.0 (Adobe Inc., San José, CA, USA) and Prism (Graphpad Software Inc., Version 5, Boston, MA, USA).

## 3. Results 

### 3.1. Reproduction and Production Parameters of the Cows

The average lactation number of the participating animals was 2.8 years. Their average amount of milk was 8928 kg/year, with an average SCC at the dry period of 139,600 cells/mL. Three of the eight cows started their dry period with a SCC above 100,000 cells/mL (Table 3). The eight cows were dried off with a teat sealant rather than an antibiotic dry-off.

### 3.2. Scoring

The physiological parameters for respiration ranged between 24–36 breaths/min and the rectal body temperature ranged between 38.0–39.0 °C. In the general examination, no clinical signs were observed besides a slightly increased breathing rate of 40 breaths/minute for one cow. Two cows were scored with a rumen filling score of “1” and six cows with a rumen filling score of “3” (Table 3). 

In the further examination, the udder contamination of the eight cows ranged between “1” and “2”; therefore, all animals’ udders were classified as clean. Two cows each had one non-intact udder quarter; therefore, no milk was collected from these quarters for further investigation. For one cow, an edema was observed in the palpation of the udder. Concerning the consistency of the milk secretion, “NAD” was scored for all cows (Table 3).

### 3.3. Conventional Bacteriological Culturing and Somatic Cell Count

In total, thirty quarters of eight animals were tested, as two quarters were not intact. Bacteriological results with “aseptic” and “NAD” were declared as negative in 26 of the 30 samples tested (86.7%). Under standard growth conditions, pathogens were detected in samples from four udder quarters of four cows and were identified as follows: Coagulase-negative *Staphylococcus* sp. (CNS; 6.7%), each in one quarter of two cows; *Corynebacterium bovis* (*C. bovis*; 3.3%) in one udder quarter of one cow; and *Streptococcus uberis* (*S. uberis*; 3.3%) in the udder quarter of one cow (Table 3). Increased SCCs above 100,000 cells/mL were detected in three animals, of which one cow displayed a SCC above 200,000 cells/mL (Table 3). Of the udder quarters with bacteriological positive samples, only one was associated with an increased SCC in the same quarter. The cytological and bacteriological results on the udder quarter level are provided in Supplementary Table S6.

### 3.4. Evaluation of the Condition of the Udder Health

Three cows—C2, C3, and C7—were classified according to the previously formulated criteria as “healthy” cows with normal secretion. Three cows—C1, C4, and C5—were diagnosed with subclinical mastitis with an increased SCC of more than 100,000 cells/mL, lacking clinical signs. Two cows—C4 and C5—displayed unspecific mastitis in one quarter without the detection of a pathogen. Three cows—C5, C6, and C8—showed signs of a latent infection due to the detection of mastitis pathogens but without an increase in the somatic cell count. At the time of investigation, no cow showed any signs of acute clinical mastitis.

### 3.5. General Results of the 16S rRNA Gene Amplicon Sequencing

The 16S rRNA gene amplicon sequencing provided results for cows C1, C5, C6, and the mock community (MC). In total, 267,308 sequences with an average of 46,051 sequences per sample (SD ± 22,411.5) were obtained. The OTU table (operational taxonomic unit, i.e., molecular species) contained 80 OTUs, and the zOTU table (zero-radius operational taxonomic units, i.e., molecular strains) contained 81 zOTUs. The raw sequencing reads and the rarefaction curves are provided in Supplementary Table S7 and Supplementary Figure S1, respectively. The DNA content in all samples was measured using a fragment analyzer at Eurofins (Eurofins Genomics GmbH; Table 4), and gel electrophoresis pictures are provided in Supplementary Figure S2.

**Figure 1:**
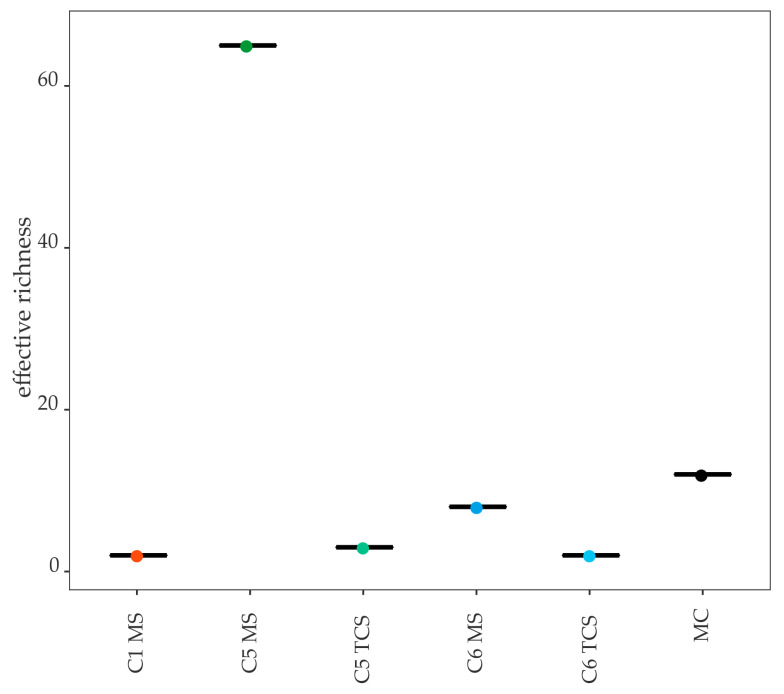
α-diversity shown as effective richness between different sample types and cows. C1, C5, C6, cows 1, 5, 6; MS, milk sample; TCS, teat canal sample; MC, mock community.

The bacterial richness for all MS and TCS was below 100 bacterial species, and the effective richness displayed even lower amounts for all samples besides the CMS of Cow 5 (Figure 1).

#### 3.5.1. Mock Community

**Figure 2:**
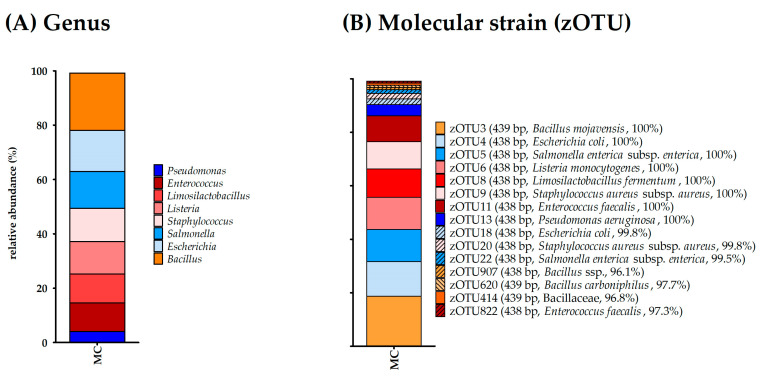
(**A**) Bacterial genera and (**B**) molecular strains obtained from 16S rRNA gene amplicon sequencing of the MC. zOTUs were identified by EzBioCloud [45]; the sequence length, the closest relative taxon, and the sequence similarity score of zOTUs are shown in the order of appearance. MC, mock community; zOTU, zero-radius operational taxonomic unit; bp, base pairs.

As a mock community, the ZymoBIOMICS Microbial Community Standard (Zymo Research Europe GmbH) was used, which contained the bacterial species *Pseudomonas aeruginosa*, *Escherichia coli*, *Salmonella enterica*, *Lactobacillus fermentum*, *Enterococcus faecalis*, *Staphylococcus aureus*, *Listeria monocytogenes*, and *Bacillus subtilis* and the two yeasts *Saccharomyces cerevisiae* and *Cryptococcus neoformans* according to the manufacturer. The DNA content of the MC displayed the highest amount of DNA by far compared to all other samples (Table 4). It was furthermore the only sample that provided some form of a band on the agarose gels (Figure S2). α-diversity analysis displayed twelve bacterial species for richness and effective richness; however, several bacterial species were represented by two or more OTUs (Table 5). Altogether, genera from all bacterial species listed by the manufacturer were detected by 16S rRNA gene amplicon sequencing, while no contamination was detected (Table 5, Figure 2).

#### 3.5.2. Negative Controls

The negative controls from the NaCl used to wet the swabs for the TCS, the DNA Stool Stabilizer (Invitek Molecular GmbH) used to stabilize the DNA from TCS, and the mock sample testing the contamination of sampling in the milking parlor during sampling all contained only minimal amounts of DNA (Table 4, Figure S2); thus, library preparation and sequencing were not possible.

### 3.6. Condition of the Udder—Healthy

For C2, C3, and C8, neither the MS nor the TCS provided DNA amounts sufficient to produce a library and allow 16S rRNA gene amplicon sequencing (Table 4). This low amount of DNA agreed with the negative results of the bacterial culture.

### 3.7. Condition of the Udder—Latent

Cow 5 is represented in both the subclinical and the latent group, and the results from the microbiome analysis are presented under Section 3.8.

**Figure 3:**
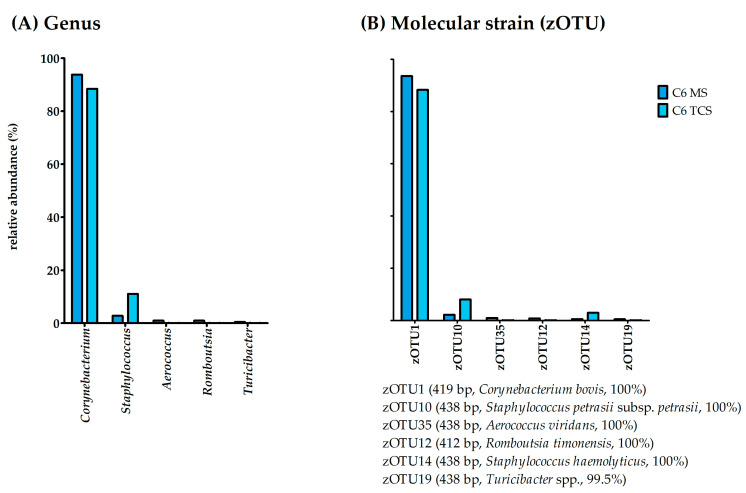
Relative abundance (%) of (**A**) bacterial species and (**B**) molecular strains for Cow 6 for MS and TCS. zOTUs were identified by EzBioCloud [45]; the sequence length, the closest relative taxon, and the sequence similarity score of zOTUs are shown in the order of appearance. MS, milk samples; TCS, teat canal samples; zOTU, zero-radius operational taxonomic unit; bp, base pairs.

The 16S rRNA gene amplicon sequencing was possible for both the MS and TCS for Cow 6. The bacterial richness was similar for both MS and TCS with 35 and 31 bacterial species, respectively. The effective richness was higher in MS (eight effective species) than in TCS (two effective species; Figure 1). The evenness for both sample types was below 0.1 for Cow 6; consequently, this sample was dominated by one or a few bacterial species. This becomes obvious when examining the genus and molecular strain composition of this cow’s MS and TCS (Figure 3).

As detected in the bacterial culture, both the MS and TCS were dominated by the genus *Corynebacterium* and the molecular strain zOTU1 (*Corynebacterium bovis*, 100% similarity). However, the 16S rRNA gene amplicon sequencing detected further molecular strains with lower abundances (Figure 3A), of which zOTU10 (*Staphylococcus petrasii* subsp. *petrasii*, similarity 100%) displayed abundances of 2.2% in MS and 8.1% in TCS (Figure 3B).

The MS and TCS samples from Cow 8 displayed low DNA amounts of 0.16 ng/µL and 0.56 ng/µL (Table 4), respectively. As described for the samples with low DNA amounts above, library preparation and thus sequencing was not successful for the MS and TCS of Cow 8. However, in the bacterial culture, CNS was detected with a very low SCC of 6000 cells/mL.

### 3.8. Condition of the Udder—Subclinical

**Figure 4:**
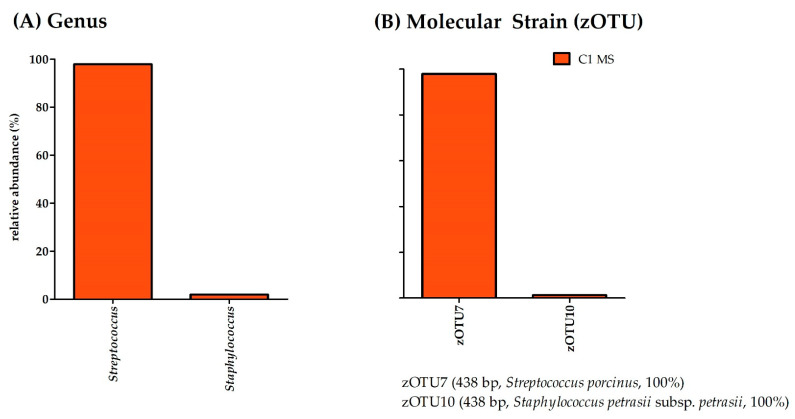
Relative abundance (%) of (**A**) bacterial species and (**B**) molecular strains for Cow 1 MS. zOTUs were identified by EzBioCloud [45]; the sequence length, the closest relative taxon, and the sequence similarity score of zOTUs are shown in the order of appearance. MS, milk sample; zOTU, zero-radius operational taxonomic unit; bp, base pairs.

The DNA content of the Cow 1 samples was 2.12 ng/µL for the MS and 0.44 ng/µL for the TCS (Table 4). Consequently, it was not possible to obtain sequencing results from the TCS. However, results for MS were available. α-diversity analysis displayed a richness of eleven bacterial species and an effective richness of two bacterial species (Figure 1). With an evenness of under 0.1 (i.e., 0.03), this CMS was again dominated by one molecular species (Figure 4).

According to the results of the bacterial culture, the MS of Cow 1 was dominated by the genus *Streptococcus*. However, unlike the results from the bacterial culture (i.e., *Streptococcus uberis*), the molecular strain dominating this sample (i.e., zOTU7) was 100% similar to *Streptococcus porcinus* (*Strep. porcinus*). With a relative abundance of over 1.0%, the molecular strain zOTU10 (*Staphylococcus petrasii* subsp. *petrasii* (*Strep. petrasii*), similarity 100%) was further detectable by molecular analysis.

In the MS and TCS from Cow 4, we measured a DNA content of 0.1 ng/µL and 0.06 ng/µL, respectively (Table 4). As described above, library preparation and thus 16S rRNA gene amplicon sequencing were not successful for these samples. This low amount of DNA agreed with the negative results of the bacterial culture, even if the SCC was increased.

**Figure 5:**
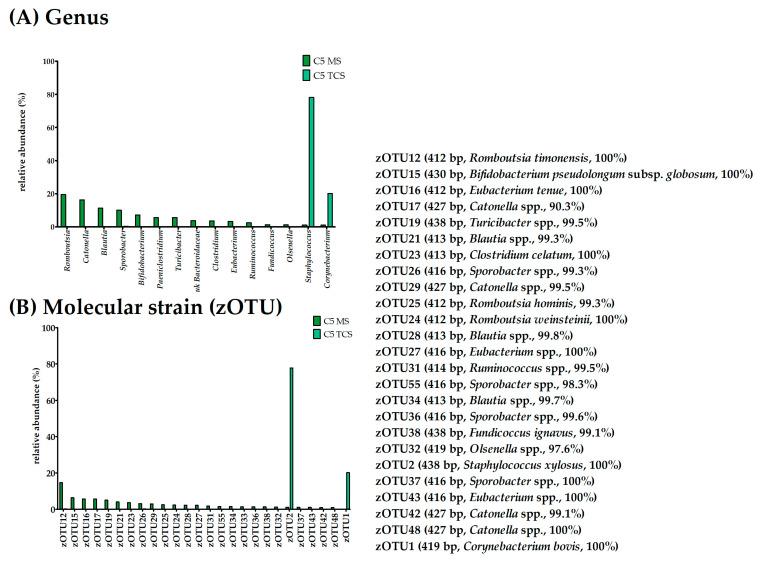
Relative abundance (%) of (**A**) bacterial species and (**B**) molecular strains for Cow 6 MS and TCS. zOTUs were identified by EzBioCloud [45]; the sequence length, the closest relative taxon, and the sequence similarity score of zOTUs are shown in the order of appearance. MS, milk samples; TCS, teat canal samples; zOTU, zero-radius operational taxonomic unit; bp, base pairs.

Both the MS and TCS of Cow 5 contained enough DNA for conducting 16S rRNA gene amplicon sequencing (Table 4). The bacterial richness in the MS displayed seventy bacterial species and sixty-five effective species, while the TCS displayed fifty-nine bacterial species and three effective species (Figure 1). The evenness was 0.59 for the MS and 0.13 for the TCS; thus, these samples had a wider distribution of species compared to those of Cow 1 and Cow 6. The results of the bacterial culture displayed coagulase-negative *Staphylococcus* sp. In the MS, we observed, unlike in all other MS, a wide variety of bacterial species with a relative abundance below 20% (Figure 5A). Interestingly, these molecular strains are species that are known to inhabit feces. Thus, it is likely that these findings represent contamination. Fitting to this assumption, the TCS from Cow 5 did not display this wide variety of species, but rather the culture-detected *Staphylococcus* genus. In the TCS samples, the genus *Staphylococcus* is represented by zOTU2 (*Staphylococcus xylosus*, similarity 100%; Figure 5B). As in all other sequencing results, we additionally found another molecular strain with a higher abundance, in this case, *Corynebacterium bovis* (zOTU1, similarity 100%; Figure 5B).

## 4. Discussion

The general analysis and the further examination of the udder have provided information on the cows’ health conditions. Only a few animals showed minor deviations from the reference parameters (Table 3), and no cow showed signs of clinical mastitis in the general and specific udder examinations, which would have excluded them from the study. Thus, the sampling of CMS as well as MS and TCS for 16S rRNA gene amplicon sequencing was performed. The results of the cytological and bacteriological examination displayed subclinical or latent mastitis for cows C1, C4, C5, C6, and C8. As all sampled cows were in the periparturient period and the immune system can be compromised during this time [21], the slight clinical signs in some animals were not surprising. In most animals, the rumen filling was scored as “3”. The rumen of two animals was less filled with a score of “1”. A low rumen filling shortly after the calving can indicate typical diseases of the early puerperium, such as mastitis or metritis [33]. The two cows with a low rumen filling score (i.e., “1”) were diagnosed with either subclinical or latent mastitis retrospectively but displayed no signs of acute clinical mastitis. Consequently, the rumen filling score might be a useful tool to detect early post-puerperal mastitis, and further examinations should be initiated before clinical signs occur. However, continuing investigation of this parameter is necessary in the future. In the further examination of the udder, all eight cows showed an udder hygiene score between “1” and “2”; thus, the udders of the animals could be described as clean [7]. Dirty udders can lead to a higher SCC and an increased risk of intramammary infection [50]. The low degree of contamination according to the udder hygiene scores correlates with the low SCC of the eight examined cows with an average of 39,000 cells/mL on the day of examination. Unfortunately, hygiene scoring of the udder has not been included in any microbiome study yet. Since the teat skin is a possible pathway for bacteria to enter the udder, variations in the composition of the bacterial community on the skin and teat cistern of dirty and clean udders should be investigated [21].

Hereafter, insights into the bacterial community of the udder using both conventional bacterial culturing as well as 16S rRNA gene amplicon sequencing are discussed. It is important to note that the authors did not expect a bacterial community in the udder of healthy cows according to the reasons discussed by Rainard et al. [19]. Therefore, methods were used to allow the maximum capture of eventual bacterial organisms in the udder, and negative controls were provided for every step. One of these methods was the pooling of the samples on the quarter level to gain enough bacterial DNA, as sequencing is not always possible and reasonable for samples with low DNA contents. Negative controls were sampled in several steps to identify possible contaminations. This is especially important in samples where low biomass is expected, as contaminations during sampling and DNA extraction provide results that rather describe the microbiome of contaminants [30]. Unfortunately, this has not been conducted for most published bovine milk microbiota studies so far [17]. The most obvious step to reduce contamination is rigorous cleaning and disinfection to reduce contamination. In this study, even though a high effort was made to work cleanly and disinfection, one MS of Cow 5 was most likely contaminated with fecal organisms. Interestingly, some of these intestine-associated bacterial species (e.g., *Ruminococcus* spp., *Bifidobacterium* spp., and *Clostridium* spp.) found in the contaminated sample are similar to some major taxa described by Oikonomou et al. [17] in their review about bovine milk microbiota. This suggests that the composition of bovine milk microbiota is strongly influenced by feces and probably does not reflect a natural community inside the mammary gland. As the bovines tend to defecate in the milking parlor, this sort of contamination cannot be ruled out and should be expected and presented as such. Nevertheless, besides this one sample, all other samples (*n* = 16) did not display any signs of contamination, and the strict hygiene management of this study allows the use of next-generation sequencing techniques even in a stable environment. As the low amount of DNA found in many samples might originate from problems during DNA extraction, a mock community was used to prove the successful extraction of DNA from bacteria that can be expected in an udder. In this work, we used the ZymoBIOMICS Microbial Community Standard (Zymo Research Europe GmbH). The extraction of all bacterial species in the mock community was successful (Figure 2); nonetheless, separation on the molecular strain level occurred, as described previously [51]. In general, it should be noted that different studies based on 16S rRNA gene amplicon sequencing are comparable to a limited extent due to the differences in sampling and sample management (e.g., DNA extraction or sequencing of different hypervariable regions) [17]. In this study, much information regarding host, environment, and management practices was provided to avoid possible misleading conclusions about the microbial origin and for a better comparison with other studies [20]. It should be further noted that this study was a preliminary test of methods to examine if we are able to find bacteria that might be members of a microbiome. Only eight clinically healthy cows were monitored by general and further specific udder examination. It would be preferable to obtain a better understanding of the SCC and CMS bacterial content before 16S rRNA gene amplicon sequencing; however, to the best of our knowledge, no such examination exists that would allow the simultaneous probing of bacterial culture and sequencing of the 16S rRNA gene amplicons from only healthy cows. Thus, it is vital to examine the existence of an udder microbiome in healthy cows using a larger study population and sampling during different phases of lactation. The planning and implementation of this study were based on conditions in practice to not only be of scientific use, but also to examine whether next-generation sequencing is applicable in a field environment and allows veterinarians a deeper insight into udder health. This study did not provide hints at the presence of a microbiome in healthy udders, while the results of CC-positive milk samples correspond to the bacterial genera and species present in the udders of cows with subclinical and latent mastitis. The cultured bacteria were always close to the top zOTUs found by sequencing in other studies, as well [52,53]. However, in addition to the bacteria detected in the culture, an additional bacterium with a lower relative abundance was discovered by sequencing. Furthermore, almost all mentioned bacteria found are potential mastitis pathogens with various levels of virulence. Some researchers believe that the detection of DNA from several types of bacteria is indicative of mastitis representing a multi-agent disease rather than being caused by a single pathogen [52,54]. Based on the results of this study, this hypothesis is not a sound basis to explain all findings. In the udders of conspicuous animals, not a multitude of bacteria was found, but mostly two zOTUs with a relative abundance that marked these bacteria as somewhat significant in a clinical context. Generally, mastitis-causing agents can be classified according to their origin. In addition, they can be divided into major and minor pathogens according to their prevalence and the severity of clinical signs [55]. *Staph. petrasii,* belonging to the coagulase-negative staphylococci (CNS), can be categorized as a minor pathogen [56]. However, to our knowledge, this pathogen has not yet been described as a mastitis-causing pathogen in cows. Before an infection with CNS, predisposing factors affecting the immune system make the udder susceptible [7]. CNS as well as *C. bovis* are found on the udder’s skin, but *C. bovis* especially has been isolated from the teat canal [7]. *C. bovis* is a minor mastitis-causing pathogen and is associated with the mammary gland. The actual origin of *C. bovis* (i.e., from milk or the teat canal) is not clear in this study, as the milk passes through the teat canal during manual sampling, and therefore, “contamination” rather than an intramammary infection is more likely [57]. According to our results from 16S rRNA gene amplicon sequencing, the bacterium was isolated in the teat canal as well as in the sample from the gland cistern, which may be an indication of emerging colonization from the teat canal. Nevertheless, the results from the MS of Cow 6 should be interpreted with caution due to the sampling method via milking tube, as a spread of *C. bovis* from the teat canal to the gland cistern cannot be excluded. In contrast, *S. uberis* is a major mastitis-causing pathogen originating mostly from the environment [55]. The general opinion about *S. uberis* as a causative agent strictly associated with the environment is debatable, and transmissions between cows can be an optional pathway for spreading [58,59]. *S. uberis* was detected in the bacterial culture from Cow 1. This finding is consistent with the results of the sequencing of the MS (i.e., *S. porcinus*). Both pathogenic species originate from the genus *Streptococcus* of the family Streptococcaceae and display a close genetic relationship [60]. Therefore, they hardly differ in terms of pathogenicity [61]. The different results from both investigations play a minor role regarding the consequences of treatment. Nevertheless, as only the V3/V4 region of the 16S rRNA gene has been sequenced, the molecular strain from 16S rRNA gene amplicon sequencing is based on the similarity of a small part of the DNA and should thus be interpreted with caution, and CC might not be able to identify the exact species. As described above, most pathogens of the genus *Streptococcus* derive from the environment, including the animals’ habitats, such as in peat or straw used as bedding material [55]. According to Metzger et al. [25], the bedding material affects the milk microbiomes even if the milk is collected directly from the gland cistern. The animals in the described study were kept on straw and showed different OTUs compared to frequently isolated OTUs (i.e., *Janthinobacterium* spp., *Enhydrobacter* spp., and Rhodocyclaceae.) from cows lying on sand, manure solids, or sawdust [25]. Contrarily, the bedding material and environment did not result in a bacterial community in the udder of cows in this study. Derakhashani et al. [21] note a lack and need for knowledge regarding the potential influence of current managerial practices according to the ten-point mastitis control program on the overall composition of the udder microbiota. There are only a couple of studies addressing this issue; studies on antibiotic drying-off and its possible influence on the milk microbiome already exist [29,62]. On the treatment of mastitis with antibiotics, only studies with *E. coli* as a mastitis pathogen are available [63,64]. Based on the results of the presented study, it would be interesting to observe the changes in sequencing results after the antibiotic treatment of the udder to investigate the elimination of bacteria. Furthermore, most of the animals in our study did not have a single-pathogen infection, and the behavior of the second pathogen after the treatment might give interesting insights into mastitis incidence. The pathogens could either be eliminated by the antibiotic treatment or, if resistant to the used antibiotic agent, this might even stimulate the growth of the second most abundant pathogen that is only detectable by sequencing and might lead to chronic mastitis that is seemingly resistant to its antibiotic therapy. Consequently, the antibiotic treatment would have eliminated one pathogen but, at the same time, prepare the way for the infection with the other pathogen.

The lack of a bacterial community in the udder of previously declared healthy cows has already been described [15,17,25]. Therefore, this study supports the assessment that there is no natural bacterial community in the healthy udder. Samples extracted directly from the gland cistern via needle incision through the udder skin display low DNA amounts, which might be associated with the cleanliness of the method [20]. Sampling directly from the gland cistern by using a needle and vacuum tube is an invasive method that requires sedation and rigorous cleaning and is therefore not suitable for routine clinical work [20]. A less invasive method using a milking tube was used for this study. This method allows sampling from the gland cistern and is less prone to contamination from the teat apex and skin compared to manual milk sampling. The method is similar to that of Friman et al. [26] or the sampling described by Vangroenweghe et al. [65]. In our work, the extraction of milk after disinfection using a milking tube resulted in a low amount of contamination while the cow’s acceptance was high, as most cows are accustomed to milking tubes during mastitis treatment and dry-off. In conclusion, this method is fitting for the extraction of cisternal milk samples, even for veterinary use.

Due to its high sensitivity and decreasing costs, sequencing is increasingly used in examining udder health. Furthermore, the inclusion of sequencing techniques in the remediation of troubled dairy herds would be beneficial. More advanced techniques such as metagenomic or whole-genome sequencing might allow the exact identification of the pathogen on the species level and might, in combination with the analysis of comparative samples from the stable (e.g., bedding, milking system, or staff), give insight into the disease incidence and pathogen origin.

## 5. Conclusions

Considering the results at the cow level, the following general conclusions can be drawn from this study. No evidence of a microbiome in the healthy udder was found in this study. This does not refer to the animals, which were conspicuous in the cytological and bacteriological examination. Here, a “bacterial community” was found, but this community consisted of only two pathogens with an abundance of higher than 1.0%, which indicates a mixed infection of the udder. The results from the CC agree mostly with the results from the molecular examination. Nonetheless, 16S rRNA gene amplicon sequencing was able to generate even more detailed insight. Consequently, next-generation sequencing might be a helpful method to understand the complexity of mastitis incidence as well as the origin of the pathogen and might thus help to eliminate possible infection sources. Additionally, this method can be used in a field environment when contamination is avoided and teats are disinfected rigorously. Researchers and clinicians should keep in mind that sequencing methods are highly sensitive and negative controls are always needed. Furthermore, the results should be questioned critically to avoid errors arising from environmental contaminations and should be interpreted in relation to the clinical parameters and the conventional culturing technique.

## Figures and Tables

**Table 1:** Overview of the scoring of the cows including general examination, further examination of the udder, and sensory examination of the milk secretion.

Subject of Examination	Examination Method	Evaluation Key	Source
General examination			
Rectal temperature	Rectal thermometer	[°C]	[32]
Breathing rate	Counting respiratory movements at the costal arch and flank	Breaths per minute	[32]
Rumen filling	Rumen filling score	1, 3, 5	[33]
Further examination of the udder			
Hygiene of the udder skin	Hygiene score	1–4	[7,34,35]
Abnormalities in texture, condition, temperature	Palpation	Yes/No	[32]
Functionality of each udder quarter	Palpation	Intact/Blind	[32]
Sensory secretion examination			
Consistency of the milk secretion	Visual evaluation	NAD, a–f	[32]

Rumen filling score, 1—low, 3—moderate, 5—well; hygiene score, 1—free of dirt, 2—slightly dirty, 3—moderately covered with dirt, 4—covered with caked-on dirt; consistency of the secretion, NAD—no abnormality detected, a—retained milk character, watery consistency, without flocs, b—retained milk character, watery consistency, small flocs, c—retained milk character, a few large flocs, d—retained milk character with many large flocs, e—milk character mostly lost, predominantly flocs, f—milk character completely reversed, instead: purulence, blood, serum, fibrin flocs, mucus.

**Table 2:** Evaluation of cytological and bacteriological results as part of mastitis categorization on the quarter level according to Fehlings et al. [38].

Quarter Level		Bacteriological Examination	
		Negative	Positive
SCC	≤ 100,000 cells/mL	normal secretion	latent infection
	> 100,000 cells/mL	unspecific mastitis	mastitis

SCC, somatic cell count.

**Table 3:** Overview of all data collected in the scoring and the cytological and bacteriological examination including the evaluation.

Cow–ID			C1	C2	C3	C4	C5	C6	C7	C8	Average	Median
**Reproduction and production parameters of the cows**												
Number of lactation instances			2	2	3	4	2	5	2	2	2.8	2
Dry period in days			64	51	55	52	59	60	62	64	58.4	59.5
Average amount of milk from previous lactations in kg			9708	9978	8436	7975	9116	10,783	9943	5483	8928	9412
**Results of the general examination**												
Rectal temperature in °C			38.4	38.6	38.8	38.5	38.5	38.0	38.5	38.4	38.5	38.5
Breathing rate in breaths per minute			22	32	22	24	36	24	24	**40**	28	24
Rumen filling (“1”, “3”, or “5”)			3	3	3	**1**	3	**1**	3	3	3	3
**Results of the further examination of the udder and the sensory secretion examination**												
Hygiene of the udder skin (“1”–“4”)			1	2	2	2	1	2	2	1	2	2
Abnormalities in texture, condition, temperature			Yes (Edema)	No	No	No	No	No	No	No		
Consistency of the secretion			NAD	NAD	NAD	NAD	NAD	NAD	NAD	NAD		
**Cytological and bacteriological results with an evaluation of the udder health**												
SCC before drying off (in 1000 cells per mL)			125	16	57	43	34	611	69	162	139.6	63.0
SCC average on seven DIM (in 1000 cells per mL)			54	20	11	50	101	26	29	22	39.0	27.4
Examinations on the quarter level on seven DIM	FL	BC	neg	neg	neg	neg	neg	neg	neg	**CNS**		
		SCC	9	14	11	**131**	36	10	26	6		
	FR	BC	neg	neg	neg	neg	neg	neg	neg	neg		
		SCC	32	21	13	10	**237**	15	38	30		
	RR	BC	***S. uberis***	neg	neg	neg	neg	neg	neg	-		
		SCC	**149**	19	12	8	46	27	26	-		
	RL	BC	neg	neg	neg	-	**CNS**	***C. bovis***	neg	neg		
		SCC	27	24	9	-	84	50	27	29		
Condition of the udder health (i.e., mastitis) *			subclinical	healthy	healthy	subclinical	subclinical, latent	latent	healthy	latent		

Bold letters display unphysiological results during the examination. * Classification according to Fehlings (2012) [38]. C1–C8, cows 1–8; Rumen filling score, 1—low, 3—moderate, 5—well; hygiene score, 1—free of dirt, 2—slightly dirty, 3—moderately covered with dirt, 4—covered with caked-on dirt; consistency of the secretion, NAD—no abnormality detected. DIM, days in milk; FL, front-left quarter; FR, front-right quarter; RR, rear-right quarter; RL, rear-left quarter; BC, results from the bacteriological culturing; SCC, somatic cell count; neg, negative; *S.*, *Streptococcus*; CNS, coagulase-negative *Staphylococcus* sp.; *C.*, *Corynebacterium* sp. Quarters without results (-) in the cytological and bacteriological examination were blind.

**Table 4:** DNA content in experimental samples.

Cow ID	Sample Type	DNA Content (ng/µL)
C1	MS	2.12
	TCS	0.44
C2	MS	0.05
	TCS	0.38
C3	MS	0.04
	TCS	0.31
C4	MS	0.10
	TCS	0.06
C5	MS	5.35
	TCS	2.22
C6	MS	1.34
	TCS	3.85
C7	MS	0.24
	TCS	0.25
C8	MS	0.16
	TCS	0.56
Mock sample		0.03
DNA stool stabilizer		0.02
Mock community		41.23
NaCl		0.59

C1–8, cow 1–8; MS, milk sample; TCS, teat canal sample.

**Table 5:** Bacterial species present in the mock community (MC) according to the manufacturer’s specifications and obtained molecular species (OTU).

Bacterial Species	Representing OTUs
*Pseudomonas aeruginosa*	OTU11
*Escherichia coli*	OTU4
*Salmonella enterica*	OTU5
*Lactobacillus fermentum*	OTU8
*Enterococcus faecalis*	OTU9
*Staphylococcus aureus*	OTU2, OTU645, OTU417
*Listeria monocytogenes*	OTU6
*Bacillus subtilis*	OTU3, OTU787, OTU462

## Data Availability

The data that support the findings of this study are only available in the Sequence Read Archive under the reference number PRJNA956443.

## Supplementary Materials

The following supporting information can be downloaded at: https://www.mdpi.com/article/10.3390/microorganisms11051311/s1, Table S1: Evaluation of rumen filling using a visual three-stage scoring system according to Donat et al. (2020) [33], modified by Zaajer und Noordhuizen (2003) [37] and Götze et al. (2019) [36]; Table S2: Udder hygiene scoring of the udder and lower leg according to Winter et al. (2009) [7], Schreiner and Ruegg (2002) [34], and Cook and Reinemann (2007) [35]; Table S3: Consistency of the secret modified according to Dirksen et al. (1998) [32]; Table S4: Obtained operational taxonomic units (OTUs); Table S5: Obtained zero-radius operational-taxonomic units (zOTUs); Table S6: Results of the cytological and bacteriological evaluation on quarter level (*n* = 30) categorized according to Fehlings et al. (2012) [38]; Table S7: Raw and denoised read stats; Figure S1: Rarefaction Curves; Figure S2: Gel electrophoresis pictures of all samples’ DNA content (*n* = 12) from (A) TCS and (B) MS.
