# Supplementary material for: Bovine Udder Health: From Standard Diagnostic Methods to New Approaches—A Practical Investigation of Various Udder Health Parameters in Combination with 16S rRNA Sequencing
Source: Microorganisms. 2023 May 17;11(5):1311. doi: 10.3390/microorganisms11051311 (PMC10221688; doi:10.3390/microorganisms11051311)
Supplement: Supplementary file 1 [file microorganisms-11-01311-s001.zip › Supplementary Tables S1, S2, S3, S6.pdf]

Table S1. Evaluation of rumen filling using a visual three-stage scoring system according to Donat et al. (2020), modified by Zaajer and Noordhuizen (2003) and Götze et al. (2019).

**Rumen filling score**	**Rumen filling**	**Appearance of the *Fossa paralumbalis*** Relation to the vertebral transverse processes	Relation to the last rib	**Shape**
1	Low	Cavitates a hand's width inside under the transverse process	Cavitates a hand's width behind it	Empty rectangle
3	Moderate	Falls about a hand's width vertically down and then bulges out	Cavitates less than a hand's width behind it	Triangle
5	Well	Transverse process hardly visible	Last rib hardly visible	Bulges out directly

Table S2. Udder hygiene scoring of the udder and lower leg according to Winter et al. (2009), Schreiner and Ruegg (2002), and Cook and Reinemann (2007).

**Score 1**	**Score 2**	**Score 3**	**Score 4**
Free of dirt, clean	Slightly dirty, 2 – 10% of the surface covered	Moderately covered with dirt, 10 – 30% of the surface covered	Covered with caked-on dirt, > 30% of the surface covered

Table S3. Consistency of the secret modified according to Dirksen et al. (1998).

**Consistency of the secret**							
score	NAD	a	b	c	d	e	f
evaluation of the consistency of the udder secretion	secretion obviously unchanged: normal milk of lactating cows and colostrum	retained milk character, watery consistency, without flocs	retained milk character, watery consistency, with small flocs	retained milk character, with a few large flocs	retained milk character with many large flocs	milk character mostly lost, predominantly flocs	milk character completely reversed, instead: purulence, blood, serum, fibrin flocs, mucus

NAD, no abnormality detected.

Table S4. Obtained operational taxonomic units (OTUs)

Excel file

Table S5. Obtained zero-radius operational-taxonomic units (zOTUs)

Excel file

Table S6. Results of the cytological and bacteriological evaluation on quarter level (*n* = 30) categorized according to Fehlings et al. (2012).

**Quarter level**		**Bacteriological examination**	
		**Negative**	**Positive**
**SCC**	**≤ 100,000 cells/mL**	*n* = 24	*n* = 3
	**> 100,000 cells/mL**	*n* = 2	*n* = 1

SCC, somatic cell count
