# Supplementary figures and images for: Bovine Udder Health: From Standard Diagnostic Methods to New Approaches—A Practical Investigation of Various Udder Health Parameters in Combination with 16S rRNA Sequencing
Source: Microorganisms. 2023 May 17;11(5):1311. doi: 10.3390/microorganisms11051311 (PMC10221688; doi:10.3390/microorganisms11051311)

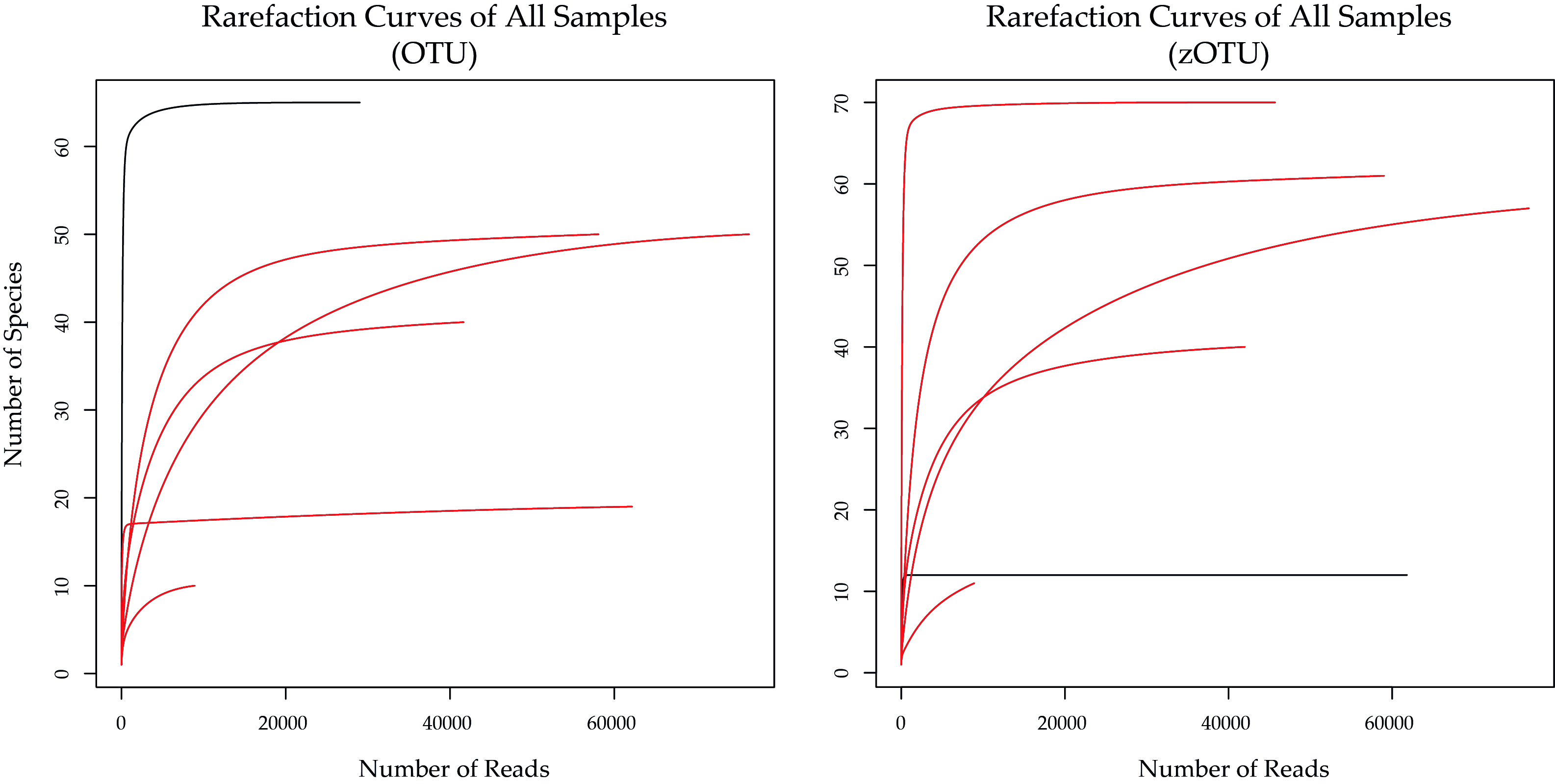

Supplement: Supplementary file 1 [file microorganisms-11-01311-s001.zip › Supplementary Figure S1.jpg]
